# Mucins Help to Avoid Alloreactivity at the Maternal Fetal Interface

**DOI:** 10.1155/2013/542152

**Published:** 2013-06-20

**Authors:** Arnela Redzovic, Gordana Laskarin, Marin Dominovic, Herman Haller, Daniel Rukavina

**Affiliations:** ^1^Department of Physiology and Immunology, Medical Faculty, University of Rijeka, B. Branchetta 20, 51000 Rijeka, Croatia; ^2^Department of Radiotherapy and Oncology, Clinical Hospital Rijeka, Kresimirova 42, 51000 Rijeka, Croatia; ^3^Division of Cardiology, Hospital for Medical Rehabilitation of the Heart and Lung Diseases and Rheumatism “Thalassotherapia”, M. Tita 188, 51410 Opatija, Croatia; ^4^Department of Obstetrics and Gynecology, Clinical Hospital, University of Rijeka, Kresimirova 42a, 51000 Rijeka, Croatia

## Abstract

During gestation, many different mechanisms act to render the maternal immune system tolerant to semi-allogeneic trophoblast cells of foetal origin, including those mediated via mucins that are expressed during the peri-implantation period in the uterus. Tumour- associated glycoprotein-72 (TAG-72) enhances the already established tolerogenic features of decidual dendritic cells with the inability to progress towards Th1 immune orientation due to lowered interferon (IFN)-**γ** and interleukin (IL)-15 expression. Mucine 1 (Muc 1) supports alternative activation of decidual macrophages, restricts the proliferation of decidual regulatory CD56^+^ bright natural killer (NK) cells, and downregulates their cytotoxic potential, including cytotoxic mediator protein expression. Removing TAG-72 and Muc 1 from the eutopic implantation site likely contributes to better control of trophoblast invasion by T cells and NK cells and appears to have important immunologic advantages for successful implantation, in addition to mechanical advantages. However, these processes may lead to uncontrolled trophoblast growth after implantation, inefficient defence against infection or tumours, and elimination of unwanted immunocompetent cells at the maternal-foetal interface. The use of mucins by tumour cells to affect the local microenvironment in order to avoid the host immune response and to promote local tumour growth, invasion, and metastasis confirms this postulation.

## 1. Introduction

The mother and conceptus are engaged in a chemical conversation throughout pregnancy [1]. Cycling endometrium provides a microenvironment in which molecules secreted by uterine cells, including glycoproteins mucin-1- (Muc 1-) and tumour-associated glycoprotein-72 (TAG-72), are transported into the uterine lumen, where they represent histotrophs required for blastocyst growth and development [2]. An active embryonic stimulus is required to initiate implantation in terms of the removal of epithelial surface glycoproteins from directly beneath the implantation site [2]. The blastocyst forms directly under uterine influence after its apposition and adhesion to the receptive endometrium [2, 3]. Cytokines, chemokines, and growth factors expressed by decidual cells, with their pleiotropic and redundant functions, are broadly involved in stimulating growth, differentiation, and the function of uterine and trophoblast cells, as well as in their mutual coordination and synchronization [2, 4]. Blois et al. [5] showed that pregnancy-specific glycoproteins influence trophoblast immune evasion. Additionally, angiogenesis in mice and its predictive value for the pregnancy outcome have been considered [5]. Recently, the broad and complex role(s) of Muc 1 and TAG-72 was evaluated at the maternal-foetal interface at the beginning of pregnancy, when the dynamic exchange of cytokine orientations was indispensable for immunoadherence [6]. However, many studies in animal and human models failed to completely elucidate mucin-mediated immunophysiological and immunopathological pathways involved in establishing pregnancy.

## 2. Alloreactivity at the Maternal-Foetal Interface

During gestation, many different mechanisms act to render the maternal immune system tolerant to the foetus. Induction of the adaptive immune response of an allograft begins with the recognition of the alloantigen by the mother's T cells. This generally occurs through 3 main processes, including the direct, indirect, and semidirect pathways, depending on the antigen presented within major histocompatibility complex (MHC) molecules [7]. However, “the first signal” by itself induces T-cell anergy, which is followed by an active immune response, representing immunomodulation in the absence of “the second signal” [8]. The second signal represents engagement of costimulatory molecules on antigen-presenting cells (APCs) with CD28 or CTLA4 on the surface of T cells, allowing selective, activating, or inhibiting T-cell responses [8]. As the second signal providers, decidual dendritic cells (DCs) as well as macrophages play very important roles in immune adjustment during pregnancy [9, 10]. Human early pregnancy decidua harbours primarily myeloid mature CD83^+^ DCs [11], a very small population of intermediate CD205^+^ DCs [12] and CD1a^+^ cells [11, 13]. In humans, the number of mature CD83^+^ cells decreases during early pregnancy compared to that in the late secretory phase, whereas immature CD209^+^ DCs dominate in the decidua [14, 15]. Decidual T cells are hyporeactive to trophoblast alloantigens [16], possibly because of their interaction with cognate immune DCs. Therefore, it has been hypothesized that antigen presentation by immature DCs induces tolerance, whereas antigen presentation by mature DCs induces immunity [17, 18]. However, mature decidual DCs induce CD4^+^ T-cell tolerance in mice [19]. Lung tissue-specific DCs under steady-state conditions are sufficiently mature to express intermediate levels of MHC class II and costimulatory molecules, but these cells polarize T cells towards the Th2 helper pathway [20].

Thus, the assumption that different maturation states or different subsets have varying tolerogenic functions can no longer be used to distinguish between the tolerogenic and immunogenic properties of DCs [21]. Additionally, freshly isolated human myeloic decidual mature DCs show a reduced capacity to produce IL-12 p70 compared to peripheral blood DCs [22]. Naïve allogeneic human CD4^+^ T cells primed with these DCs led to a higher percentage of Th2 cells than peripheral myeloid DCs [22]. However, the assumption that decidual DCs have tolerogenic functions must be further investigated because natural killer (NK) cells and macrophages may stimulate DCs to secrete IL12 and may cause further induction of Th1 cytokine production by lymphocytes, resulting in apoptosis of trophoblast cells and a Th1-type immune bias of abortion [23]. This finding strongly suggests that the microenvironment is a potent, or perhaps the most important, modulator of DC function [24], but the substances responsible for these actions, including mucins, remain to be elucidated.

## 3. Accessory Regulators of Alloreactivity

**Figure 1:**
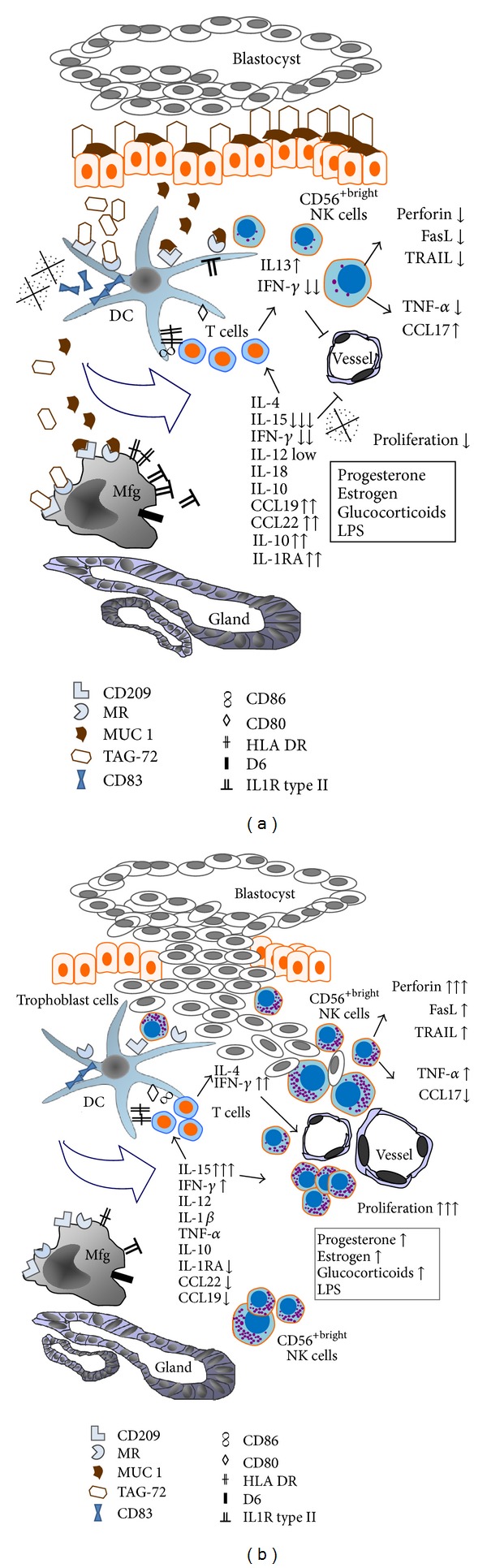
Proposed model for antigen-presenting cells and decidual lymphocytes interactions at the maternal-foetal interface in the presence (a) and absence (b) of mucin-1- (Muc 1-) and tumour-associated glycoprotein-72 (TAG-72). The functions of dendritic cells (DCs) and macrophages (Mfgs) may be influenced by Muc 1 and TAG-72 that bind to the mannose receptor (MR) and CD209. TAG-72-shaped DCs may produce less CD83, resulting in lower proliferation and selective apoptosis of cognate cytotoxic T cells to allow survival of Th2-oriented T cells with low production of IFN-gamma (IFN-*γ*), attracted by CC chemokine ligand- (CCL-)19 and CCL22. Mfgs in the presence of TAG-72 produced higher levels of interleukin (IL)-10 and IL-1 receptor antagonist (IL-1RA), but significantly decreased levels of IL-12 and CCL3, support a Th2 bias. Muc 1-shaped Mfgs increased IL-1 receptor type II (IL-1R type II) expression, whereas a D6 decoy, CD80, CD86, and human leukocyte antigen (HLA)DR remain relatively unchanged. Muc 1-shaped Mfgs and TAG-72-treated DCs appear to decrease IL-15 production and cannot support the proliferation of CD56 bright NK cells and expression of cytotoxic mediators. Low IFN-*γ* expression by TAG-treated DCs does not support decidual vessel remodelling. During normal eutopic implantation, removing surface epithelial glycoproteins (b) allows antigen-presenting cells to support mild proinflammatory reactions by increasing IL-15 and IFN-*γ* production and amplifying NK cells, which are rich in cytotoxic mediators.

Distinct profiles of cytokine and chemokine secretion by appropriately stimulated DCs induce and orientate T cells [25], representing “the third signal” in T-cell activation. DCs use T-cell-mediated responses by stimulating “naïve” T cells via the Th2-promoting cytokine IL-10 to induce tolerance at the maternal-foetal interface and via the Th1-promoting cytokine IL-12 to stimulate T-cell activity [10], in the absence of appropriate tolerogenic stimuli. Prostaglandin E2, supported by progesterone, polarizes the maturation of myeloid DCs into Th2-promoting DCs, and transforming growth factor beta promotes tolerogenic DCs [26]. Both mediators are present at the maternal-foetal interface during normal pregnancy and may be utilized by DCs to regulate decidual health. Progesterone enhances the expression of leukaemia inhibitory factor (LIF) [27], which is one of the most important factors involved in fertilization and implantation [28]. In humans, maximum expression of LIF occurs between 19 and 25 days of the menstrual cycle, corresponding to the opening of the “implantation window” [2]. However, significant thinning of the glycoprotein layer (glycocalyx) beneath the trophoblast implantation site, including disappearance of its constituent Muc 1, is indispensable for successful implantation in many species [29]. Muc 1 acts as a physical barrier between the cell surface and external environment, thus protecting cells from microorganisms, toxins, or proteolytic factors, as well as preventing trophoblast invasion with equal efficiency [30, 31]. We recently demonstrated the absence of tumour-associated TAG-72 in uterine decidua of normal and pathological human pregnancies (nonembryonic pregnancy and missed abortion) at the implantation sites although TAG-72 was present in epithelial cells at and away from the tubal implantation site of an ectopic pregnancy [32]. Moreover, our recent *in vitro* studies confirmed the interaction between Muc 1 [33] and TAG-72 [32] with APCs at the maternal-foetal interface [32]. Our experiments suggested that they participate in the maturation of CD1a^+^ DCs and macrophages and critically influence their phenotype, cytokine and chemokine production, and functions, as shown in Figure 1(a). This method of controlling immune reactions, including trophoblastic invasion, occurs at the maternal-foetal interface [34].

## 4. Regulation of Mucin Expression

Mucins are a family of heavily glycosylated proteins [35]. Numerous groups of transmembrane mucins include Muc 1, which harbours a transmembrane domain, a short cytoplasmic tail, and an extensive extracellular domain [33]. Mucins are ubiquitously present in diverse mucous membranes and are localized at the apical surface of polarized epithelial cells in the lungs, stomach, intestines, and eyes [36, 37].

Most studies examining the role of mucins in reproduction have focused on human Muc 1 because of its predominant expression in the human endometrium [38]. Muc 1 is present at the apical surface of epithelial cells in the endometrium throughout the menstrual cycle [39]. Human Muc 1 expression is high during the peri-implantation period [40]. It is likely the first molecule that the embryo encounters before adhering to the endometrium [30, 41] (Figure 1(a)). In the uterus, Muc 1 suppresses the interaction between the implanting embryo and maternal endometrial adhesion molecules, thus creating a barrier to implantation in humans [30] as well as many other mammalian species [31]. This theory has been confirmed by experimental data that show that mice lacking the gene for Muc-1 have a persistently receptive endometrium [38, 42]. Its removal is necessary for successful implantation in humans [43]. Indeed, Muc-1 is not present beneath the embryo implantation site [29, 30]. It is thought that embryos send paracrine signals and participate in Muc-1 clearance [43]. Muc 1 may participate in selectin-dependent interactions in the uterus, thus promoting cell-cell interactions in some contexts [44]. Muc 1 can be also removed from the site of implantation because of paracrine signals from local milieu [43], including different bacterial and viral products [45]. It could be overexpressed by hypoxia at the maternal-foetal interface and contributes to hypoxia-driven angiogenesis, as it was seen previously in the pancreatic cancer cells [46]. TNF-*α* is a cytokine secreted by endometrium and blastocyst, which probably mediated binding of nuclear factor kappa β to its binding site in the promoter of Muc-1 gene [45]. On the other hand, TNF-*α* leads to the removal of Muc-1 in human uterine epithelial cells by increasing expression of sheddase TACE (tumour necrosis factor-alpha-converting enzyme)/ADAM17 (a disintegrin and metalloprotease-like 17) [47]. Membrane-type matrix metalloprotease (MT1-MMP) also contributes eliminating the external part of Muc-1 molecule independently of TACE/ADAM17, and its expression increases during the receptive phase in the endometrial biopsy [48]. Furthermore, immunohistochemistry demonstrated the colocalization of MUC-1 and MT1-MMP in human uterine epithelium during implantation [48].

Moreover, Muc 1 is transcriptionally regulated by oestrogen, progesterone, and glucocorticoids [49, 50]. Sex hormones control gene transcription of Muc 1 either by direct interaction with the Muc 1 promoter or indirectly by stimulating or suppressing other transcription factors in the uterus [49]. Progesterone increases the synthesis and secretion of Muc 1 at the systemic level during early pregnancy, but cells—both beneath and immediately adjacent to attached embryos—do not contain detectable levels of Muc 1, whereas the cells further away in the epithelial layer remain unaffected [49, 50]. Uterine tissue macrophage-derived factors (LIF) and cytokines (IL-1β) promote uterine receptivity by regulating surface glycan structures in epithelial cells [51]. Furthermore, Shyu et al. showed that Muc 1 mRNA and Muc 1 protein levels increase with gestational stage during human pregnancy, primarily because of Muc 1 expression of trophoblast cells [52].

Osteen et al. observed TAG-72 in the normal postovulatory, secretory phase endometrium, but not in the proliferative phase [53]. TAG-72^+^ cells were rarely observed in the lower uterine segment using immunohistochemistry [53]. We recently showed that TAG-72 was not present in the decidual tissue of healthy and eutopic pathological early pregnancies, including missed abortion and blighted ovum [54]. In contrast, TAG-72 was present in the tubal mucosa at and away from the implantation site during ectopic tubal pregnancy, but not in the uterine mucosa of the same woman [54]. To our knowledge, in the recent scientific literature there is little data that speaks about TAG-72 regulation; however, TAG-72 appears to be hormonally dependent. Estradiol can inhibit TAG-72 expression, whereas progesterone does not directly stimulate TAG-72 [55]. Additionally, TAG-72 downregulates oestrogen and progesterone receptors in endometriotic lesions [55]. Both autocrine and paracrine mechanisms are involved in regulating TAG-72 expression [56].

Glycoproteins, Muc 1 [30, 40] and TAG-72 [53, 54], are present in peri-implantation period in uterus and participate certainly in highly controlled process of trophoblast invasion, which resembles tumor invasion by deep decidual invasion, modifying of cellular morphology, and an epithelial-to-mesenchymal transition [3, 57]. Muc 1 overexpression suppresses extravilious trophoblast invasion mainly via modulating β1-integrin signaling in severe preeclamptic placentas [58]. β1-integrin-silenced cells show a defective activation of the epidermal growth factor receptor-signaling cascade, leading to decreased *in vitro* proliferation, impaired migration, and invasive behavior [58]. However, the modification of cellular glycosylation is a common phenotypic change of cancer cells that mainly affects the outer part of glycans, leading to the expression of tumour-associated carbohydrate antigens [59]. The transmembrane mucins, in particular, are overexpressed and aberrantly glycosylated in most cases of adenocarcinoma and are also associated with constitutive activation of a growth factor signalling, invasive proliferation of tumors, possibility of metastatic spread, and activation of a programme of tumor cell repair and survival (immortality) [3, 59]. The tumor mucins interact with monocytes, dendritic cells, and T and NK cells supporting anti-inflammatory and tolerogenic immune response toward tumor cells, favoring in that way the tumor growth with a patient's poor outcome [3, 59].

## 5. Mucins Suppress Alloreactivity at the Maternal-Foetal Interface

Trophoblast cells express mucins throughout gestation and thereby contribute to increased mucin concentrations at the maternal-foetal interface during pregnancy [60]. Whether Muc 1 affects decidual NK and T-cell functions during later pregnancy is unknown. *In vitro* Muc 1 competes with the PAM-1 monoclonal antibody, which is directed towards the carbohydrate recognition domain of the mannose receptor (MR) [25], followed by binding and internalization of MR in early decidual CD14^+^ cells in a dose-dependent manner [33]. This finding presents a possible mechanism for Muc 1 to influence the phenotype and functional properties of decidual CD14^+^ cells in *in vivo*, as illustrated in Figure 1(a). In *in vitro *experiments, Muc 1 stimulated macrophages to increase the surface expression of IL1-R type II (Figure 1(a)), which binds to IL-1 but does not transduce signals in the cell, quenching the IL-1 proinflammatory signal [61]. In contrast, a D6 decoy for proinflammatory chemokines, as well as costimulatory CD80 and CD86 molecules, HLA-DR and CD83, remains relatively unchanged after Muc 1 stimulation [33]. Muc 1 significantly decreases IL-15 in decidual macrophages, whereas IFN-*γ*, IL-18, IL-10, CCL3, and CCL17 are essentially unchanged [33].

Immature decidual CD1a^+^ cells express CD209 [15] and a high number of MRs on the surface of single cells [62]. MR contains 8 and CD209 contains 7 extracellular carbohydrate recognition domains [63], indicating that these cells can bind to highly glycosylated molecules such as TAG-72 [64] (Figure 1(a)). Indeed, TAG-72 competes with mannan, which is a primordial ligand for the carbohydrate recognition domain [64], and it binds MR and CD209 on decidual CD1a^+^ cells *in vitro *[32]. This binding is important because ligands binding the carbohydrate recognition domain of MR can activate an anti-inflammatory and a tolerogenic response in monocyte-derived DCs [25] such as decidual DCs [32]. Indeed, after TAG-72 stimulation, decidual CD1a^+^ cells show significantly decreased CD83 molecules, likely due to its active secretion [65]. CD83 molecules have tolerogenic properties owing to the significantly reduced DC-mediated T-cell stimulation [65], which may contribute to immune tolerance at the maternal-foetal interface in the presence of TAG-72 (Figure 1(a)).

## 6. Effects of Mucins on Decidual T-Cell Functions

Hiltbold et al. showed that DCs present various glycosylated or nonglycosylated forms of Muc 1 using MHC class I molecules [66]. The efficiency of processing and the resulting strength of CD8^+^ T-cell activities inversely correlated with the degree of glycosylation of the antigen [66]. Agrawal et al. also suggested that Muc-1-derived peptides are processed and presented in the context of MHC class I molecules on the surface of tumour cells [67]. CD8^+^ T cells could detect Muc 1 peptides associated with MHC class I. Most Muc 1 epitopes did not contain a consensus motif for a particular MHC class I allele and bound with low “affinity,” compared with known high-affinity peptides [68]. MHC-restricted antigen recognition of mucins indicates that a given T-cell will recognize a peptide antigen only when it is bound to a host body's self-MHC molecule [68]. Normally, T cells are stimulated only in the presence of self-MHC molecules; hence, the antigen is recognized only when peptides are bound to these self-MHC molecules. At the maternal-foetal interface, CD8^+^ T-cells are nearly depleted [68]. Magarian-Blander et al. showed that direct recognition of the Muc 1 peptide epitope by a T-cell receptor in the absence of presentation by MHCs induces a partial signal that is completed by further interactions with other receptor/ligand pairs on the surface of the CTL and their target cells [69].

TAG-72-treated decidual CD1a^+^ cells were unable to stimulate the proliferation of syngeneic decidual T cells [32], mostly of the CD45^+^ phenotype [70] (Figure 1(a)), although they significantly stimulated the proliferation of allogeneic, naïve cord blood T cells [32]. Furthermore, allogeneic T-cells cocultured with monocyte-derived DCs, which were matured using an antimannose receptor monoclonal antibody of the PAM-1 clone *in vitro*, initially proliferated but later became anergic and behaved as suppressor/regulatory cells [25]. Thus, poor proliferation that is observed upon rechallenge with cognate TAG-72-treated CD1a^+^ DCs suggests some degree of decidual T-cell tolerance. This finding agrees with the previous finding that TAG-72-treated CD1a^+^ cells decreased CD83 expression, which is considered a DC-activation marker [32] and is responsible for specific support of the expansion of newly primed naïve CD8^+^ T-cells and long-term survival of antigen-specific T cells by inducing proliferation and inhibiting apoptosis [71].

Furthermore, TAG-72-treated CD1a^+^ cells decreased IFN-*γ* production in syngeneic decidual and allogeneic cord blood T cells, even in the presence of lipopolysaccharide (LPS) [32] (Figure 1(a)). However, IL-4 is not likely to be increased in decidual T cells after close contact with TAG-72-primed CD1a^+^ DCs [32]. However, intracellular IL-4 expression was increased in allogeneic cord blood T cells, even in the presence of TAG-72 and LPS pretreated CD1a^+^ cells [32]. This powerful tolerogenic feature of TAG-72 is not observed in freshly isolated decidual T cells, which appear to be oriented towards the Th2 pathway.

Accordingly, PAM-1-treated monocyte-derived DCs could not polarize Th1 effector cells and did not secrete proinflammatory chemokines, CXCL10 and CCL19 [25]. In contrast, they produced large amounts of anti-inflammatory CCL22 and CCL17 chemokines [72]. These findings emphasize the anti-inflammatory properties of CD1a^+^ cells treated with ligands for the carbohydrate recognition domain, particularly TAG-72, in terms of its interaction with T cells. Additionally, mucins can potentiate selective survival of naïve T cells, which may be modified using mucins [39].

## 7. Effects of Mucins on Decidual NK Cell Functions

The addition of Muc 1 in a suspension of decidual mononuclear cells substantially reduced the percentage of IL-15- and IFN-*γ*-expressing CD14^+^ cells [33], as illustrated in Figure 1. Through contact with MUC-1-treated macrophages, decidual cognate NK cells proliferated less efficiently and showed significantly decreased expression of perforin, Fas Ligand, and tumour necrosis factor-related apoptosis-inducing ligand (TRAIL) based on decreased IL-15 levels in decidual CD14^+^ cells in the presence of MUC 1 [33] (Figure 1(a)). These NK cells are likely inefficient in the control of trophoblast invasion owing to their low numbers and weak cytotoxic potential. However, in the presence of Muc 1, trophoblast invasion does not occur at the eutopic implantation site. At the implantation site in tubal ectopic pregnancy, TAG-72 is present and appears to disturb trophoblast growth control because of diminished intracellular IL-15 and IFN-*γ* cytokine production in DCs [32]. The lower proportion of IL-15-expressing CD1a^+^ DCs isolated from the tubal implantation site than that isolated from the lining of the uterus of the same woman [32] supports the hypothesis and may explain deeper trophoblast invasion in the tubal wall during ectopic pregnancy; this may also be the main reason for tubal rupture and tubal pregnancy termination [73]. Similarly, tumour-associated macrophages isolated from human ovarian cancer express MR, which can bind TAG-72 to modulate cytokine production towards an unwanted immune-suppressive profile with an increased IL-10, not IL-12, and decreased Th1-attracting chemokine CCL3 expression [74]. Furthermore, TAG-72 inhibits lipopolysaccharide-mediated intracellular signalling cascades in tumour-associated macrophages induced by infection, leading the orientation of T cells with no experience, toward a Th2 response [75]. This result suggests a mechanism of alternative activation, which allows faster tumour progression by promoting tumour cell invasion, migration, and metastasis [75]. It may also explain why higher expression of TAG-72 occurs in tumour tissues during advanced tumour stages compared to early stages in ovarian [76], breast [77], and gastric [78] tumours.

In the absence of tolerogenic mucin stimulation [25, 79] at the eutopic implantation site, DCs appear to support mild proinflammatory reactions through IL-15 and IFN-*γ* production [32], as shown in Figure 1(b). IL-15-shaped decidual NK cells allow trophoblast infiltration during normal pregnancy, but they can also kill these cells in the presence of IL-15, causing termination of pregnancy [80], likely through perforin- and granulysin-mediated cell killing involving the necrotic and apoptotic pathways [81]. Perforin and granulysin expression is abundant in freshly isolated normal human decidual NK cells [82]. These apoptotic molecules can be upregulated upon cocultivation with DCs and macrophages [83], which are the main sources of IL-15. Decidual NK cells proliferate and secrete more IFN-*γ*, which plays critical roles in angiogenesis, immune modulation at the implantation site, and maintenance of the decidual (maternal) component of the placenta [84].

## 8. Conclusion

During pregnancy in mammalian maternal tissues, immunocompetent cells are in direct and intimate contact with trophoblast cells of the foetoplacental unit. Successful survival of the foetal allograft can be explained using numerous immunoregulatory mechanisms acting at the maternal-foetal interface. According to our data, the main roles of Muc 1 and TAG-72 include regulation of maturation as well as expression of chemokines and cytokines by decidual APCs. Their effects are primarily mediated by C-type lectin endocytic receptors (CD209 and MR), which direct Th2 immune responses and alternative activation of APCs [85]. Thus, decidual mucins restrain strong anti-inflammatory local milieu. Similarly, studies examining tumour models have shown that mucin overexpression, aberrant intracellular localization, and changes in glycosylation are consistently associated with stronger invasion [86]. The removal of mucins during the first trimester of pregnancy is necessary to create a moderate “inflammatory” microenvironment, successful implantation, and tissue remodelling.
